# Using the Wild Bootstrap to Quantify Uncertainty in Mean Apparent Propagator MRI

**DOI:** 10.3389/fninf.2019.00043

**Published:** 2019-06-12

**Authors:** Xuan Gu, Anders Eklund, Evren Özarslan, Hans Knutsson

**Affiliations:** ^1^Division of Medical Informatics, Department of Biomedical Engineering, Linköping University, Linköping, Sweden; ^2^Center for Medical Image Science and Visualization, Linköping University, Linköping, Sweden; ^3^Division of Statistics and Machine Learning, Department of Computer and Information Science, Linköping University, Linköping, Sweden

**Keywords:** bootstrap, diffusion MRI, MAP-MRI, RTOP, NG, PA, uncertainty

## Abstract

**Purpose:** Estimation of uncertainty of MAP-MRI metrics is an important topic, for several reasons. Bootstrap derived uncertainty, such as the standard deviation, provides valuable information, and can be incorporated in MAP-MRI studies to provide more extensive insight.

**Methods:** In this paper, the uncertainty of different MAP-MRI metrics was quantified by estimating the empirical distributions using the wild bootstrap. We applied the wild bootstrap to both phantom data and human brain data, and obtain empirical distributions for the MAP-MRI metrics return-to-origin probability (RTOP), non-Gaussianity (NG), and propagator anisotropy (PA).

**Results:** We demonstrated the impact of diffusion acquisition scheme (number of shells and number of measurements per shell) on the uncertainty of MAP-MRI metrics. We demonstrated how the uncertainty of these metrics can be used to improve group analyses, and to compare different preprocessing pipelines. We demonstrated that with uncertainty considered, the results for a group analysis can be different.

**Conclusion:** Bootstrap derived uncertain measures provide additional information to the MAP-MRI derived metrics, and should be incorporated in ongoing and future MAP-MRI studies to provide more extensive insight.

## 1. Introduction

Mean apparent propagator (MAP) MRI is a diffusion-weighted MRI framework for accurately characterizing and quantifying anisotropic diffusion properties, at large as well as small levels of diffusion sensitivity (Özarslan et al., 2013). Consequently, it has been demonstrated that MAP-MRI can capture intrinsic nervous tissue features (Özarslan et al., 2013; Avram et al., 2016; Fick et al., 2016b). Some novel features of the diffusion process can be characterized by MAP-MRI, including the return-to-origin probability (RTOP), non-Gaussianity (NG), and propagator anisotropy (PA). It has already been shown that MAP-MRI metrics can be potential biomarkers of brain microstructure, demonstrated using synthetic data, *ex-vivo* marmoset brain data (Özarslan et al., 2013) as well as *in-vivo* healthy human brain data (Avram et al., 2016; Fick et al., 2016b). Moreover, in a transgenic rat model of Alzheimer's disease, it was found that MAP-MRI metrics can be sensitive to age-dependent neuronal demise (Fick et al., 2016a). The utility of MAP-MRI metrics as biomarkers has also been demonstrated for axonal remodeling after ischemic stroke (Brusini et al., 2015) and they have been employed for distinguishing pathological (after stroke) and healthy subjects (Brusini et al., 2016; Obertino et al., 2016). Clearly, researchers have already started using MAP-MRI metrics as clinical biomarkers, due to their potential sensitivity and specificity for various diseases. However, it is still not clear how high the uncertainty is for different MAP-MRI derived metrics.

### 1.1. Why Study Uncertainty?

To use MAP-MRI metrics as clinical biomarkers, it is necessary to estimate the metric itself as well as its uncertainty. Without an uncertainty measure it is difficult to know if a subject, or a specific brain region, for example has a lower non-Gaussianity, or if it is lower by random chance. For group analyses, an uncertainty for each subject and voxel can be used to downweight outliers or subjects with more uncertain estimates (e.g., due to more severe head motion). MAP-MRI is based on a number of parameters and settings, such as the maximum order of the Hermite basis functions, how the initial tensor is estimated and the resolution of the propagator. An uncertainty measure can be used to select the settings that result in the lowest uncertainty. Similarly, it becomes possible to investigate how much the uncertainty is increased when the amount of diffusion data is reduced (to decrease MR scanner time). Furthermore, an uncertainty measure can be used for comparing different softwares and preprocessing pipelines, as a better pipeline should result in a lower uncertainty. MAP-MRI is becoming more and more popular, and has been implemented in several software packages, e.g., Dipy (Garyfallidis et al., 2014) and Tortoise (Pierpaoli et al., 2010; Irfanoglu et al., 2017). For the mentioned reasons, it is of great value to study the uncertainty of MAP-MRI derived metrics.

### 1.2. Bootstrap

Bootstrap is a non-parametric statistical technique, based on data resampling, used to quantify uncertainties of parameters (Efron, 1992). Bootstrap can be used regardless of the estimation method, in contrast to other methods that are only applicable to linear least-squares (Sjölund et al., 2018). Bootstrap has been widely used in diffusion tensor imaging (DTI) to study uncertainty associated with DTI parameter estimation (Pajevic and Basser, 2003; Heim et al., 2004; Chung et al., 2006; Yuan et al., 2008; Vorburger et al., 2012, 2016). The repetition bootstrap method requires multiple measurements per gradient direction to perform resampling (Heim et al., 2004). For most clinical and research applications, it is more interesting to obtain a higher number of gradient directions, since diffusion parameter estimation can be more precise with high angular resolution diffusion imaging (HARDI). To be able to use bootstrap for diffusion data with many gradient directions, instead of repetitions of the same direction, residual bootstrap can be used with the assumption that the error terms have constant variance. The wild bootstrap (Wu, 1986) is suited when data are heteroscedastic (i.e., have non-constant variance), and it is also valid for non-parametric regression (Ferraty et al., 2010; Sidik and Jonkman, 2016).

Implementation of the repetition bootstrap (Jones, 2003; Chung et al., 2006; Yuan et al., 2008), residual bootstrap (Chung et al., 2006; Vorburger et al., 2012), and wild bootstrap (Chung et al., 2006; Whitcher et al., 2008; Polders et al., 2011; Vorburger et al., 2012, 2016) have already been reported for DTI for single-shell data. To the best of our knowledge, there are no studies about bootstrap for multi-shell diffusion data, or for the original MAP-MRI formulation. The uncertainty of Laplacian-regularized MAP-MRI was investigated in Sjölund et al. (2018), but this version of MAP-MRI does not guarantee positivity of the propagator. Furthermore, the Bayesian approach used in Sjölund et al. (2018) is only efficient for studying uncertainty of linear MAP-MRI metrics, such as RTOP.

For the standard diffusion tensor model, the violation of constant variance comes from the common log-transformation of the diffusion signal. MAP-MRI requires diffusion data from several shells (corresponding to different b-values), and the variance differs between the shells due to the difference in signal to noise ratio. We therefore used the wild bootstrap to provide estimates of uncertainty for MAP-MRI derived quantities, for physical phantom data (SPARC) (Ning et al., 2015) and for human brain data (HCP-MGH) (Van Essen et al., 2013).

The rest of the paper is organized as follows. Section 2 describes the background theory, for MAP-MRI and bootstrap. Section 3 describes the data used in this study (phantom data and Human Connectome Project data) and presents the technical details of the implementation and data processing. Section 4 contains results for the phantom data and Human Connectome Project data. Finally, discussion and conclusion are presented in section 5.

## 2. Theory

### 2.1. MAP-MRI

We start by reviewing the fundamentals of the MAP-MRI model, since the theory is required to introduce the different MAP-MRI metrics, for which we estimate the uncertainty. The MAP-MRI approach uses a functional basis to represent the 3D diffusion signal with as few assumptions as possible. It then analytically reconstructs the 3D diffusion propagator by only assuming a short gradient pulse approximation. In this way, it accurately estimates the diffusion propagator in the presence of both non-Gaussian diffusion and crossing tissue configuration. We will study three q-space indices: Return-To- Origin (RTOP), non-Gaussianity (NG), and propagator anisotropy (PA), which in theory describe the volume of a cylindrical pore, the ratio between the Gaussian and non-Gaussian components of the signal and the anisotropy of the 3D diffusion propagator, respectively.

The three-dimensional q-space diffusion signal attenuation *E*(**q**) is expressed in MAP-MRI as

(1) $$\begin{array}{l} E\left(\text{q}\right)=\overset{N_{\text{max}}}{\underset{N=0}{\sum }}\underset{\left\{n_{1},n_{2},n_{3}\right\}}{\sum }a_{n_{1}n_{2}n_{3}}\Phi_{n_{1}n_{2}n_{3}}\left(\text{A},\text{q}\right), \end{array}$$

where Φ_*n*_1_*n*_2_*n*_3__(**A**, **q**) are related to Hermite basis functions and depend on the second-order tensor **A** and the **q**-space vector **q**. **A** can be taken to be the covariance matrix of displacement, defined as

(2) $$\begin{array}{l} \text{A}=2{\text{R}}^{T}\text{D}\text{R}t_{d}=\left(\begin{array}{ccc} u_{x}^{2} & 0 & 0\\ 0 & u_{y}^{2} & 0\\ 0 & 0 & u_{z}^{2} \end{array}\right), \end{array}$$

where **R** is the transformation matrix whose columns are the eigenvectors of the standard diffusion tensor **D**, and *t*_*d*_ is the diffusion time. The non-negative indices *n*_*i*_ are the order of Hermite basis functions which satisfy the condition *n*_1_ + *n*_2_ + *n*_3_ = *N*, and *N*_max_ is the maximum order and it is even-valued. The q-space vector **q** is defined as **q** = γδ**G**/2π, where γ is the gyromagnetic ratio, δ is the diffusion gradient duration, and **G** determines the gradient strength and direction. The diffusion propagator, a 3-dimensional probability density function, is the three-dimensional inverse Fourier transform of *E*(**q**), and can be expressed as

(3) $$\begin{array}{l} P\left(\text{r}\right)=\overset{N_{\text{max}}}{\underset{N=0}{\sum }}\underset{\left\{n_{1},n_{2},n_{3}\right\}}{\sum }a_{n_{1}n_{2}n_{3}}{\text{Ψ}}_{n_{1}n_{2}n_{3}}\left(\text{A},\text{r}\right), \end{array}$$

where Ψ_*n*_1_*n*_2_*n*_3__(**A**, **r**) are the corresponding basis functions in displacement space **r**. The number of coefficients for MAP-MRI is given by

(4) $$\begin{array}{l} N_{\text{coef}}=\frac{1}{6}\left(\frac{N_{\text{max}}}{2}+1\right)\left(\frac{N_{\text{max}}}{2}+2\right)\left(2N_{\text{max}}+3\right). \end{array}$$

The MAP-MRI basis functions, Φ_*n*_1_*n*_2_*n*_3__(**A**, **q**) in **q**-space and Ψ_*n*_1_*n*_2_*n*_3__(**A**, **r**) in displacement **r**-space, are given by

(5) $$\begin{array}{l} \Phi_{n_{1}n_{2}n_{3}}\left(\text{A},\text{q}\right)=\phi_{n_{1}}\left(u_{x},q_{x}\right)\phi_{n_{2}}\left(u_{y},q_{y}\right)\phi_{n_{3}}\left(u_{z},q_{z}\right), \end{array}$$

(6) $$\begin{array}{l} {\text{Ψ}}_{n_{1}n_{2}n_{3}}\left(\text{A},\text{r}\right)=\psi_{n_{1}}\left(u_{x},x\right)\psi_{n_{2}}\left(u_{y},y\right)\psi_{n_{3}}\left(u_{z},z\right), \end{array}$$

with Özarslan et al. (2008)

(7) $$\begin{array}{l} \phi_{n}\left(u,q\right)=\frac{i^{-n}}{\sqrt{2^{n}n!}}e^{-2\pi^{2}q^{2}u^{2}}H_{n}\left(2\pi \;uq\right), \end{array}$$

(8) $$\begin{array}{l} \psi_{n}\left(u,x\right)=\frac{1}{\sqrt{2^{n+1}\pi n!}u}e^{-x^{2}/2u^{2}}H_{n}\left(x/u\right), \end{array}$$

where *H*_*n*_(*x*) is the *n*th order Hermite polynomial. Equation (1) can be written in matrix form (with error term added on the right side) as

(9) $$\begin{array}{l} \text{y}=\text{Q}\text{a}+\varepsilon , \end{array}$$

where **y** is a vector of *T* signal values, **Q** is a *T* × *N*_coef_ design matrix formed by the basis functions Φ_*n*_1_*n*_2_*n*_3__(**A**, **q**), **a** contains the parameters to estimate, and ε is the error. The coefficients **a** can be obtained by solving the following quadratic minimization problem,

(10) $$\begin{array}{l} \underset{\text{a}}{\min}\|\text{y}-\text{Q}\text{a}{\|}^{2},\;\text{K}\text{a}\geq 0,\;1^{T}\text{K}\text{a}\leq 0.5, \end{array}$$

where **0** and **1** are vectors with elements 0 and 1, respectively. The rows of the constraint matrix **K** are the basis functions Ψ_*n*_1_*n*_2_*n*_3__(**A**, **r**) evaluated on a uniform Cartesian grid in the positive *z* half space. The first constraint enforces non-negativity of the propagator, and the second one limits the integral of the probability density (propagator) to a value no greater than 1.

Zero displacement probabilities include the return-to-origin probability (RTOP), and its variants in 1D and 2D: the return-to-plane probability (RTPP), and the return-to-axis probability (RTAP), respectively. Return-to-origin-probability, *P*(**r**), is the probability for water molecules to undergo no net displacement. In terms of MAP-MRI coefficients through the expression it is defined as

(11) $$\begin{array}{l} RTOP=\frac{1}{\sqrt{8\pi^{3}|\text{A}|}}\overset{N_{\text{max}}}{\underset{N=0}{\sum }}\underset{\left\{n_{1},n_{2},n_{3}\right\}}{\sum }{\left(-1\right)}^{N/2}a_{n_{1}n_{2}n_{3}}B_{n_{1}n_{2}n_{3}}, \end{array}$$

where

(12) $$\begin{array}{l} B_{n_{1}n_{2}n_{3}}=K_{n_{1}n_{2}n_{3}}\frac{{\left(n_{1}!n_{2}!n_{3}!\right)}^{1/2}}{n_{1}!!n_{2}!!n_{3}!!}, \end{array}$$

and *K*_*n*_1_*n*_2_*n*_3__ = 1 if *n*_1_, *n*_2_, and *n*_3_ are all even and 0 otherwise. If we consider a population of isolated pores, with the assumptions that the diffusion gradients are infinitesimally short and the diffusion time is sufficiently long, it can be shown that (Özarslan et al., 2013)

(13) $$\begin{array}{l} <V>\;=RTOP^{-1}, \end{array}$$

which indicates that the reciprocal of the RTOP is the statistical mean pore volume. The non-Gaussianity (NG) and the propagator anisotropy (PA), respectively, measures the dissimilarity between the propagator and its Gaussian and isotropic parts, according to Özarslan et al. (2013)

(14) $$\begin{array}{ll} NG & =\sqrt{1-\frac{a_{000}^{2}}{\sum_{n=0}^{N_{max}}\sum_{\left\{n_{1},n_{2},n_{3}\right\}}a_{n_{1}n_{2}n_{3}}^{2}}}, \end{array}$$

(15) $$\begin{array}{ll} PA & =\sqrt{1-\frac{{\left(\sum_{n=0}^{N_{max}}\sum_{\left\{n_{1},n_{2},n_{3}\right\}}a_{n_{1}n_{2}n_{3}}o_{n_{1}n_{2}n_{3}}\right)}^{2}}{\left(\sum_{n=0}^{N_{max}}\sum_{\left\{n_{1},n_{2},n_{3}\right\}}a_{n_{1}n_{2}n_{3}}^{2}\right)\left(\sum_{m=0}^{N_{max}}\sum_{\left\{m_{1},m_{2},m_{3}\right\}}o_{m_{1}m_{2}m_{3}}^{2}\right)}}. \end{array}$$

where *o*_*m*_1_*m*_2_*m*_3__ are the MAP-MRI coefficients of its isotropic part.

### 2.2. Bootstrap

Repetition (regular) bootstrap requires multiple measurements per gradient direction, and for each gradient direction the measurements are sampled with replacement over-and-over again to characterize the uncertainty of the diffusion derived metrics (Heim et al., 2004). However, nowadays it becomes clinically more feasible to have scan protocols with a large number of gradient directions (Jones, 2004), instead of having more than one measurement per direction.

Alternatives to repetition bootstrap are model-based bootstrap approaches, such as the residual bootstrap and the wild bootstrap. Residual bootstrap relies on the assumption that the residuals are independent and identically distributed (i.i.d); the sample diffusion data are generated by randomly sampling with replacement from the residuals. Wild bootstrap is designed for heteroscedastic data, that is when the constant variance assumption is violated. In the case of the diffusion tensor model (Basser et al., 1994), it is known that the log-transform leads to non-constant variance (Wegmann et al., 2017). Therefore, the residuals are weighted by a heteroscedasticity consistent covariance matrix estimator and random samples are drawn from the auxiliary distribution (Davidson and Flachaire, 2008). The wild bootstrap can be defined as Whitcher et al. (2008)

(16) $$\begin{array}{l} y_{i}^{*}={\left(\text{Q}\hat{\text{a}}\right)}_{i,\cdot }+\sqrt{\frac{T}{T-N_{coef}}}u_{i}\varepsilon_{i},\;i=1,\cdot \cdot \cdot \;,T, \end{array}$$

where ${\left(\text{Q}\hat{\text{a}}\right)}_{i,\cdot }$ is the *i*th row of the product of **Q** and $\hat{\text{a}}$, $\hat{\text{a}}$ is the solution of the quadratic minimization problem in Equation (10), and ε_*i*_ is the *i*th residual of the original regression model $\hat{\varepsilon }=\text{y}-\text{Q}\hat{\text{a}}$, and *u*_*i*_ is a random sample drawn from the Rademacher distribution, i.e.,

(17) $$\begin{array}{l} u_{i}=\{\begin{array}{c} 1,\;\text{with probability }0.5,\\ -1,\;\text{with probability }0.5. \end{array} \end{array}$$

Solving the quadratic minimization problem for ${\text{y}}^{*}=\left[y_{1}^{*},\cdot \cdot \cdot \;,y_{T}^{*}\right]$ will produce a bootstrap estimate of the coefficients a^*^. Repeating these steps for some fixed large number *N*_*B*_, resampling and estimation, builds up a collection of coefficients ${\text{a}}_{1}^{*},\cdot \cdot \cdot \;,{\text{a}}_{N_{B}}^{*}$ called the bootstrap distribution, from which some MAP-MRI scalar indices can be calculated. Summary statistics from this empirical distribution can be used to describe the original parameter estimate. Here the sample statistic $\hat{\theta }$ is an estimate of the true unknown θ (such as the noise-free RTOP of the voxel) using the original data **y**, and ${\hat{\theta }}^{*}$ is the bootstrap replication of $\hat{\theta }$. The bootstrap-estimated standard error of $\hat{\theta }$ is simply the standard deviation of the *N*_*B*_ replications, i.e.,

(18) $$\begin{array}{l} {\text{STD}}_{\theta }=\sqrt{\frac{1}{N_{B}-1}\overset{N_{B}}{\underset{n\;=\;1}{\sum }}{\left[{\hat{\theta }}^{*}\left(n\right)-{\bar{\theta }}^{*}\right]}^{2}}, \end{array}$$

where ${\bar{\theta }}^{*}=\frac{1}{N_{B}}\overset{N_{B}}{\underset{n\;=\;1}{\sum }}{\hat{\theta }}^{*}\left(n\right)$. In this paper we use the standard deviation for comparing the dispersion of parameters.

## 3. Data and Methods

In this section we first detail the diffusion data used in the following study. We used phantom data with known fiber configuration to quantify its MAP-MRI metrics uncertainty. We also used human subjects data from the Human Connectome Project. To perform group comparisons of MAP-MRI metric maps, the data of four subjects were transformed to a standard space. Finally, to verify the wild bootstrap method, we manually added Gaussian noise of different standard deviation to the MAP-MRI fitted signal.

### 3.1. SPARC Phantom Data

We used data from the Sparse Reconstruction Challenge for Diffusion MRI (SPARC dMRI) hosted at the 2014 CDMRI workshop on computational diffusion MRI (Ning et al., 2015). The data were acquired from a physical phantom with known fiber configuration. The phantom is made of polyfil fibers of 15 μm diameter (Moussavi-Biugui et al., 2011). It provides a mask to indicate the number of fiber bundles crossing in each voxel. In two-fiber voxels, the fiber bundles are crossing at a 45 degree angle with isotropic diffusion outside. The voxels that are masked by 0 have no fibers and are not considered. Three sets of data are acquired with b-values of 1,000, 2,000, and 3,000 s/mm^2^, using 20, 30, and 60 gradient directions per shell for the three datasets respectively (hereinafter referred to as SPARC-20, SPARC-30, and SPARC-60). The gold-standard data was obtained by acquiring 81 gradient directions at b-values of 1,000, 2,000, 3,000, 4,000, and 5,000 s/mm^2^ averaged over 10 repetitions, resulting in 405 measurements (hereinafter referred to as SPARC-Gold). All datasets include one measurement with *b*_0_. The data has dimension 13 × 16 × 406 and resolution 2 × 2 × 7 mm. The diffusion time and pulse separation time are δ = Δ = 62 ms.

### 3.2. Human Connectome Project MGH Adult Diffusion Data

We used the MGH adult diffusion dataset from the Human Connectome Project (HCP) (Setsompop et al., 2013). Data were collected from 35 healthy adults scanned on a customized Siemens 3T Connectom scanner with 4 different b-values (1,000, 3,000, 5,000, and 10,000 s/mm^2^). The data has already been preprocessed for gradient non-linearity correction, motion correction and eddy current correction (Glasser et al., 2013). The data consists of 40 non-diffusion weighted volumes (b = 0), 64 volumes for b = 1,000 and 3,000 s/mm^2^, 128 volumes for b = 5,000 s/mm^2^ and 256 volumes for b = 10, 000 s/mm^2^, which yields 552 volumes of 140 × 140 × 96 voxels with an 1.5 mm isotropic voxel size. The diffusion time and pulse separation time are δ = 12.9 ms and Δ = 21.8 ms. The HCP-MGH data also contains high-resolution T1 images of 256 × 256 × 276 voxels with an 1.0 mm isotropic voxel size.

Data used in the preparation of this work were obtained from the Human Connectome Project (HCP) database (https://ida.loni.usc.edu/login.jsp). The HCP project (Principal Investigators: Bruce Rosen, M.D., Ph.D., Martinos Center at Massachusetts General Hospital; Arthur W. Toga, Ph.D., University of Southern California, Van J. Weeden, MD, Martinos Center at Massachusetts General Hospital) is supported by the National Institute of Dental and Craniofacial Research (NIDCR), the National Institute of Mental Health (NIMH) and the National Institute of Neurological Disorders and Stroke (NINDS). HCP is the result of efforts of co-investigators from the University of Southern California, Martinos Center for Biomedical Imaging at Massachusetts General Hospital (MGH), Washington University, and the University of Minnesota.

### 3.3. Simulated Data

We used the MAP-MRI fitted signal of subject MGH-1010 with manually added Gaussian noise to verify the wild bootstrap method. The ratio between mean signal of *b*_0_ voxels within the brain and the standard deviation of noise was used as the SNR measure. Three levels of Gaussian noise with SNR = 10, 5, and 2 were added to the MAP-MRI fitted signal of subject MGH-1010.

### 3.4. Comparison of Phantom Data and Human Data

The SPARC data is based on a physical phantom with a crossing angle of 45 degree, using polyfil fibers with a diameter of 15 μm. h The diffusion outside of the fiber bundles is isotropic, which can at best mimic diffusion in the extracellular space. Fibers were wound wet onto the spindles to generate anisotropic water diffusion. The diffusion time is 62 ms. The SNR can be estimated as the ratio between mean signal and the standard deviation of noise. The SNR of *b*_0_ for one-fiber voxels was estimated to be 45, 52, 52, 59 for SPARC-20, SPARC-30 and SPARC-60, SPARC-Gold, respectively. The SNR of *b*_0_ for two-fiber voxels was estimated to be 23, 26, 26, 30 for SPARC-20, SPARC-30 and SPARC-60, SPARC-Gold, respectively. The HCP-MGH data was acquired from healthy adults. The diffusion time is 21.8 ms. We used PIESNO (Koay et al., 2009) to identify noise voxels and estimate standard deviation. The SNR of *b*_0_ for white matter was estimated to be 34, 40, 32, 38 for subject MGH-1003, 1005, 1007, and 1010, respectively. The SNR of *b*_0_ for gray matter was estimated to be 54, 66, 57, 63 for subject MGH-1003, 1005, 1007, and 1010, respectively.

### 3.5. Methods

Diffusion tensor fitting, MAP-MRI fitting and bootstrap sampling were implemented using C++ and the code is available on Github^1^. The initial tensor fitting was performed with data with b-values < 2,000 s/mm^2^ using weighted least squares. To impose the constraint of positivity of the propagator, we sample *P*(**r**) in a 21 × 21 × 11 grid, resulting in 4851 points. Here the last dimension is only sampled on its positive axis as the propagator is antipodally symmetric. We use the Gurobi Optimizer (Gurobi Optimization, 2016) to solve the quadratic optimization problem. The Open Multi-Processing (OpenMP) (Dagum and Menon, 1998) framework is used to run the analysis for many voxels in parallel. MAP-MRI fitting and bootstrap sampling are computationally expensive, due to the large number of MAP coefficients, constraints in the quadratic minimization problem and repeating the analysis 500–5,000 times. We use a computer with 512 GB RAM and two Intel(R) Xeon(R) E5-2697 2.30 GHz CPUs. Each of the two CPUs has 18 cores (36 threads), which makes it possible to run the analysis for 72 voxels in parallel.

In order to perform voxel-level group comparisons of diffusion-derived metric maps, the diffusion data must be transformed to a standard space. The transformation between MNI standard space and diffusion space was achieved in three separate steps. First, the non-diffusion volume was registered to the T1 volume using the *FSL* (Jenkinson et al., 2012) function *epi*_*reg*. Second, the T1 volume was non-linearly registered to the MNI152 T1 2 mm template using the *FSL* function *fnirt* (Andersson et al., 2007). Third, the two transformations were combined, to transform the diffusion data to MNI space. The statistics analysis was performed in MATLAB (R2016b, The MathWorks, Inc., Natick, Massachusetts, United States).

## 4. Results

In this section we present several experiments that investigate the uncertainty of MAP-MRI metrics using simulated data, phantom data, and human diffusion data. We begin by first showing the diffusion scalar maps (FA, MD, RTOP, NG, and PA) of the SPARC data, to study the fiber configurations of the phantom. Following this, we present results investigating the impact of diffusion acquisition scheme, i.e., number of shells and number of measurements per shell. We then present the uncertainty of RTOP, NG, and PA for HCP data, focused one axial slice of four subjects. Finally, we assess the impact of preprocessing by comparing the uncertainty of RTOP for raw and preprocessed data.

It has previously been reported that including terms up to order 6 (*N*_max_ in Equation 1) was found to yield a sufficient level of detail in propagators from diverse brain regions (Fick et al., 2016b). It is recommended to use order 4 for data with few shells, according to Hutchinson et al. (2017). All further analyses of MAP-MRI parameters described in this paper use *N*_max_ = 4 for SPARC data and *N*_max_ = 6 for HCP data, if not specified otherwise. With the help of OpenMP and the Gurobi Optimizer, we are able to perform the MAP-MRI fitting for SPARC-30 using a *N*_max_ of 6 within 1.5 s, which is 33 times faster than its counterpart in Fick et al. (2016b). Computation time can be an issue when the number of bootstrap samples is large. The implementation of MAP-MRI in Fick et al. (2016b) takes 55 s to fit MAP-MRI (*N*_max_ = 6) for the SPARC-30 data (which has only one slice of 208 voxels), that is 55 × 1, 000 s = 15 h for 1,000 bootstrap samples. Our implementation makes it possible to collect the same number of samples within 0.4 h, using 40 CPU threads.

### 4.1. SPARC

**Figure 1:**
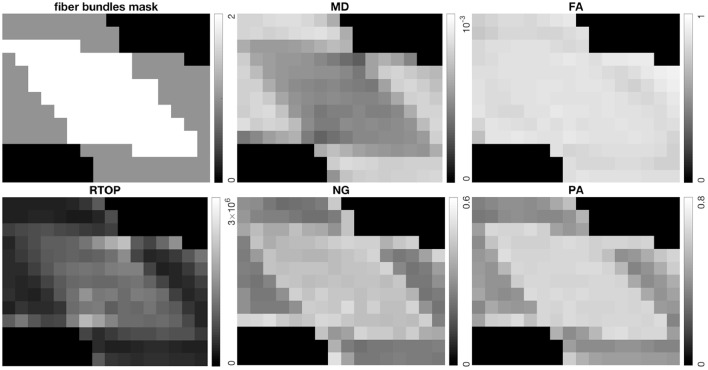
Top left: The fiber bundles mask, top middle: Mean Diffusivity (MD) (mm^2^/s), top right: Fractional Anisotropy (FA), bottom left: RTOP (mm^−3^), bottom middle: NG, bottom right: PA for SPARC-Gold. It can be seen that MD, RTOP, NG, and PA differ for different fiber configurations, while FA shows less contrast in fibrous areas.

**Figure 2:**
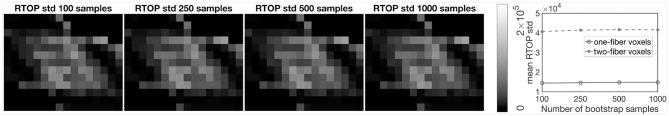
Standard deviation of RTOP for SPARC-Gold, using 100, 250, 500, and 1,000 bootstrap samples. SPARC-Gold has five shells and 81 measurements for each shell. Mean standard deviation plots of RTOP are shown for one-fiber and two-fiber voxels, radial order *N*_max_ = 4.

Figure 1 shows the scalar maps of the fiber bundles mask, fractional anisotropy (FA), mean diffusivity (MD), RTOP, NG, and PA. The values in the fiber bundles mask indicate the number of fiber bundles in each voxel. The voxels masked by 0 are referred as empty area. The construction of the physical phantom is described in Moussavi-Biugui et al. (2011). The MD, RTOP, NG, and PA clearly show different diffusivities in two-fiber areas and single-fiber areas. To investigate whether 1,000 bootstrap samples are adequate, we present the standard deviation maps of RTOP for SPARC-Gold using 100, 250, 500, and 1,000 bootstrap samples in Figure 2. Using only 100 bootstrap samples slightly underestimates the standard deviation, while 500 bootstrap samples results in standard deviations close to using 1,000 samples.

**Figure 3:**
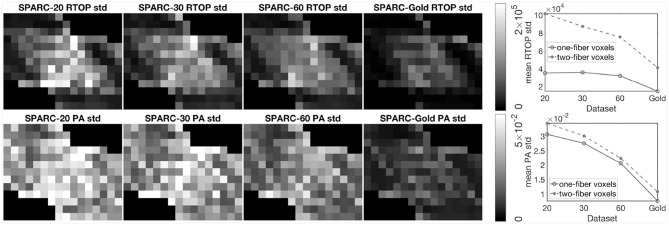
Standard deviation of RTOP and PA for SPARC-20, SPARC-30, SPARC-60, and SPARC-Gold, using 1,000 bootstrap samples. SPARC-20, SPARC-30, and SPARC-60 have three shells and 20, 30, 60 measurements for each shell. SPARC-Gold has five shells and 81 measurements for each shell. Mean standard deviation plots of RTOP and PA are shown for one-fiber and two-fiber voxels, radial order *N*_max_ = 4.

To investigate the impact of diffusion acquisition scheme (number of shells and number of measurements per shell) on the uncertainty of MAP-MRI metrics, 1,000 bootstrap samples of RTOP and PA were generated for SPARC-20, SPARC-30, SPARC-60, and SPARC-Gold using radial order *N*_max_ = 4. Standard deviation of the 1,000 RTOP and PA samples are shown in Figure 3. Alongside the standard deviation maps, the mean standard deviation of RTOP and PA are shown for one-fiber and two-fiber voxels. SPARC-20, SPARC-30, and SPARC-60 have the same three shells but different number of measurements per shell: 20, 30, and 60, respectively. SPARC-Gold has five shells and 81 measurements per shell. In general, the standard deviation maps and the mean standard deviation plots demonstrate that the uncertainty of RTOP and PA depend on the diffusion acquisition scheme. It can be noticed that the five-shell dataset SPARC-Gold always demonstrates a lower uncertainty for both RTOP and PA, compared with the three-shell datasets. For RTOP, increasing the number of measurements per shell from 20 to 60 has different impacts on the one-fiber and two-fiber voxels. However for PA, the uncertainty for both types of voxels decreases equally as the number of measurements per shell increases. Thus, it can be concluded that the number of measurements per shell have different degrees of impact on the uncertainty of RTOP and PA and voxels with different fiber structures.

**Figure 4:**
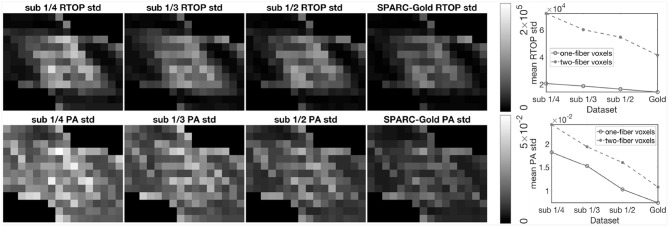
Standard deviation of the RTOP and PA for SPARC-Gold with 1/4, 1/3, 1/2, and all measurements per shell, using 1,000 bootstrap samples. SPARC-Gold has five shells and 81 measurements for each shell. Sub-data 1/4, 1/3, and 1/2 have 20, 27, and 40 measurements, respectively. Standard deviation plots of RTOP and PA are shown for one-fiber and two-fiber voxels for SPARC-Gold and each subsampled dataset.

To further investigate the impact of number of measurements per shell, we sub-sampled the SPARC-Gold data by keeping 1/4, 1/3, and 1/2 of the measurements per shell. One thousand bootstrap samples of RTOP and PA were generated for each sub-sampled dataset, results are shown in Figure 4. For all sub-sampled datasets and both MAP-MRI metrics, the two-fiber voxels give a lower uncertainty than the one-fiber voxels. The conclusions can be again confirmed, that is the RTOP uncertainty of one-fiber voxels does not greatly depend on the number of measurements per shell. Secondly, the PA uncertainty has a stronger dependence on the number of measurements per shell. The mean standard deviation of RTOP and PA are linearly correlated with the number of measurements per shell.

### 4.2. HCP-MGH

**Figure 5:**
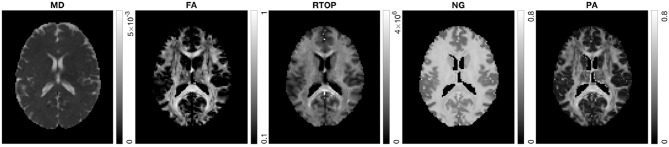
MD, FA, RTOP, NG, and PA for subject MGH-1010, slice 45. CSF voxels were removed prior to data analysis.

In the following section, we present results from HCP-MGH data. Figure 5 shows MD, FA, RTOP, NG, and PA of a slice from subject MGH-1010. The RTOP map revealed higher values in white matter (especially in the corpus callosum) than in gray matter. The RTOP tissue contrast may reflect overall restrictions and cellularity better than does the MD. NG is high in white matter and low in gray matter, and homogenous for both tissue clusters. PA measures diffusion anisotropy based on the angular dissimilarity of the propagator relative to its isotropic counterpart for MAP-MRI and Gaussian (DTI) approximations, respectively.

#### 4.2.1. Uncertainty of RTOP, NG, and PA

**Figure 6:**
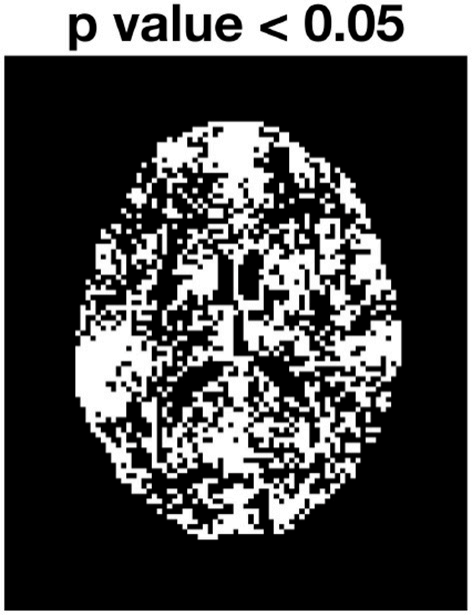
White test for subjects MGH-1010, slice 45. White voxels have residuals where the variance changes over time.

The bootstrap approach to be applied depends on if the residuals have a constant variance (homoscedasticity). A common test for heteroscedasticity is the White test (White, 1980). We applied the white test to the residual in every voxel, the voxels that survive an (uncorrected) significance level of *p* = 0.05 are shown as white voxels in Figure 6. Clearly, most of the voxels have residuals with a heteroscedastic variance, which means that the wild bootstrap is the appropriate method to use.

**Figure 7:**
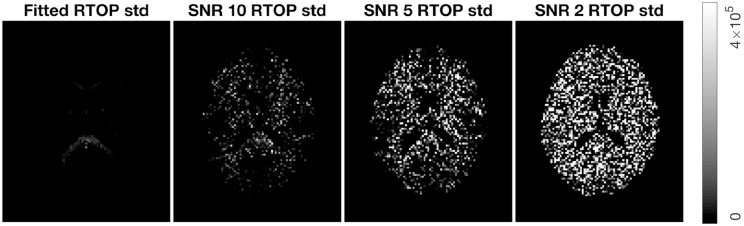
Standard deviation of RTOP for MAP-MRI fitted signal of subjects MGH-1010, slice 45, using 500 bootstrap samples. Gaussian noise of SNR = 10, 5, and 2 were added.

To verify wild bootstrap's ability of quantifying uncertainty, we added Gaussian noise with SNR = 10, 5, and 2 to the MAP-MRI fitted signal. Wild bootstrap was then applied to calculate the standard deviation of RTOP. The results are presented in Figure 7. It shows that uncertainty is increased as the SNR decreased.

**Figure 8:**
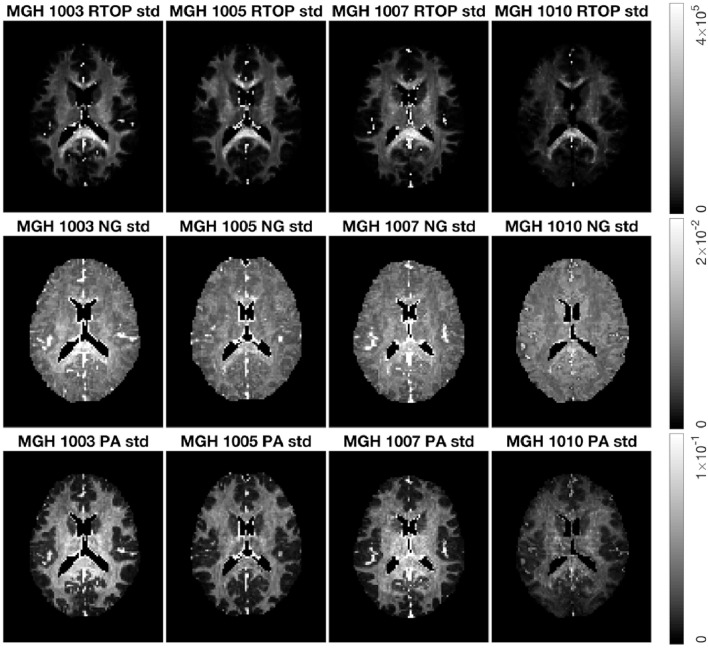
Standard deviation of RTOP, NG, and PA for subjects MGH-1003, 1005, 1007, 1010, slice 45, using 500 bootstrap samples. CSF voxels were removed prior to data analysis.

Figure 8 shows the standard deviation of RTOP, NG, and PA for subjects MGH-1003, 1005, 1007, and 1010. There are two main clusters of voxels in the RTOP standard deviation maps wherein the white matter areas generally appear hyperintense, while the gray matter areas make up the lower intensity regions. A portion of the white matter regions shows higher standard deviation values for RTOP, most notably in the splenium of the corpus callosum. Within the corpus callosum, the splenium part shows a higher standard deviation than the genu and body of the corpus callosum. The standard deviation maps for RTOP, NG, and PA show marked differences across subjects. It is interesting to note that the RTOP standard deviation for subject MGH-1010 is clearly lower compared to the other three subjects.

**Figure 9:**
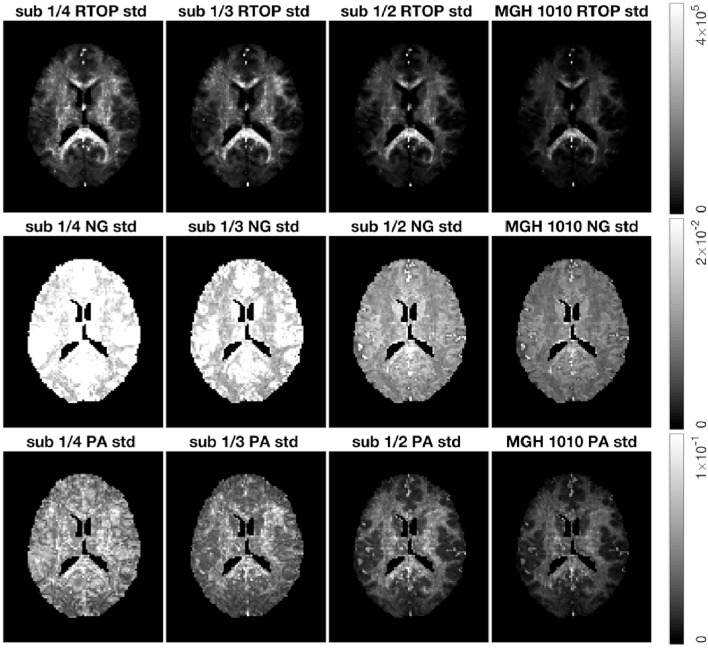
Standard deviation of the RTOP, NG, and PA for subject MGH-1010 with 1/4, 1/3, 1/2, and all measurements per shell, using 500 bootstrap samples. A lower number of measurements clearly leads to a higher uncertainty. CSF voxels were removed prior to data analysis.

As for the phantom data, we sub-sampled the MGH-1010 data by keeping 1/4, 1/3, and 1/2 of the measurements per shell. Five hundred bootstrap samples of RTOP, NG, and PA were generated for each subsampled dataset, results are shown in Figure 9. A clear decrease in the uncertainty of the three MAP-MRI metrics can be observed for a larger number of measurements per shell. Both NG and PA are greatly affected by the measurements per shell, while RTOP is less vulnerable. The PA standard deviation changes from a noisy map to an anatomically meaningful map as the measurements per shell increases.

#### 4.2.2. Group Analysis

**Figure 10:**
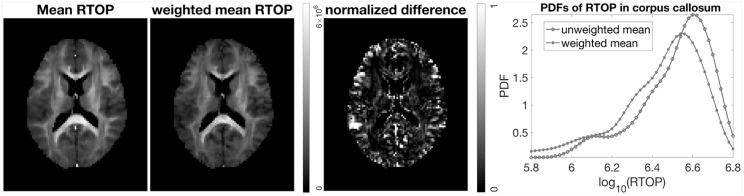
Mean and weighted mean of RTOP for subjects MGH-1003, 1005, 1007, and 1010, using 500 bootstrap samples. The normalized difference was calculated as the difference divided by the weighted mean of RTOP. Probability density function of RTOP was estimated for the corpus callosum, and is shown to the right.

It is common to perform group studies using diffusion MRI, to for example find differences between healthy controls and subjects with some disease. One of the most common scalar measures for group analysis is fractional anisotropy, calculated from the diffusion tensor, which for example has been shown to be sensitive to diffuse axonal injuries in mild traumatic brain injury (Shenton et al., 2012; Eierud et al., 2014). Existing approaches for group analyses such as TBSS (Smith et al., 2006), do not incorporate the uncertainty, and can for example not downweight subjects with a high degree of head motion. Incorporation of uncertainty can result in better group analyses. For example, as shown in Figure 8, subject MGH-1010 shows a lower uncertainty for the RTOP maps compared to the other three subjects. When calculating the mean RTOP map for a group, subjects like MGH-1010 which have lower uncertainty should contribute more in the weighted mean RTOP map. A weighted mean can be calculated as (Sjölund et al., 2018)

(19) $$\begin{array}{l} \bar{x}=\frac{\sum_{n\;=\;1}^{N}\left(w_{n}x_{n}\right)}{\sum_{n\;=\;1}^{N}w_{n}}, \end{array}$$

where $w_{n}=1/\sigma_{n}^{2}$ and σ_*n*_ is the standard deviation for subject *n*. Instead of each voxel subject contributing equally to the final mean, subjects with higher standard deviation contribute less “weight” than others. A comparison between the mean RTOP and the weighted mean RTOP is presented in Figure 10. The weighted mean for example downweights an outlier close to the posterior cingulate. A notable difference can be found in the corpus callosum, which has a relatively high uncertainty in the RTOP map. In Figure 10, also presented is a comparison of the unweighted and the weighted probability density distributions of RTOP for the corpus callosum. Subjects with a higher uncertainty will be downweighted, which can lead to a skew of the mean RTOP distribution.

#### 4.2.3. Impact of Artifacts

**Figure 11:**
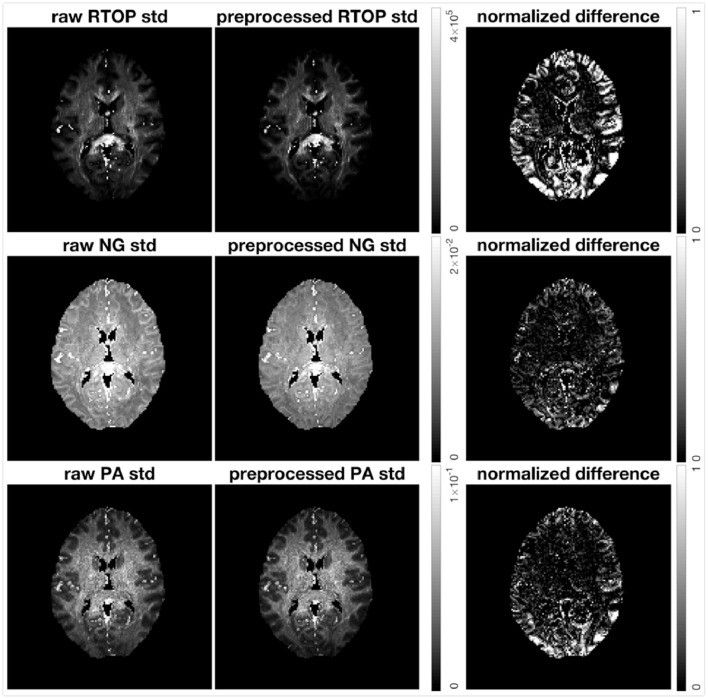
Standard deviation of RTOP, NG, and PA of preprocessed (gradient non-linearity correction, head motion correction, and eddy current correction) and raw (only gradient non-linearity correction) data for subject MGH-1010, using 500 bootstrap samples. The normalized difference was calculated as the difference divided by the RTOP, NG, or PA of the of preprocessed data. Clearly, preprocessing results in a lower uncertainty.

The HCP-MGH data provided as “preprocessed” have already been corrected for gradient non-linearity, head motion and eddy currents. The data provided as “raw” were only corrected for gradient non-linearity. To investigate how the imaging artifacts, e.g., head motion and eddy currents, affect the uncertainty of MAP-MRI metrics, we generated 500 bootstrap samples for both preprocessed and raw data. Results for subject MGH-1010 are shown in Figure 11. All three scalar maps (RTOP, NG, PA) show consistent patterns; preprocessing reduces the uncertainty. The boundaries of the brain are more vulnerable to imaging artifacts and show a larger uncertainty, especially for RTOP and PA. This is likely related to the head motion present in the raw data.

## 5. Discussion

Researchers have previously applied bootstrap methods to the linear regression used to fit the diffusion tensor model. We have extended this technique to a more sophisticated diffusion model to quantify the uncertainty of its derived metrics. We used the wild bootstrap technique to evaluate the uncertainty of the MAP-MRI derived metrics, using physical phantom data as well as human brain data. While uncertainty was previously investigated for linear MAP MRI measures (Sjölund et al., 2018), we here also show uncertainty estimates for non-linear measures (such as NG and PA), which cannot as easily be obtained using the Bayesian approach proposed by Sjölund et al. (2018).

The experiments are divided into two sections: one dealing with uncertainty estimation for physical phantom data, in which four sets of data are collected using different number of measurements and different b-values, and the other dealing with uncertainty estimation for human brain data of four healthy subjects scanned using the same protocol. The uncertainty originates from measurement noise, physiological noise, head motion, and is influenced by a wide range of parameters, many of which are difficult or impossible to fully model, such as signal-to-noise-ratio. It is important to note that all acquisition parameters which influence the SNR of the diffusion signals, such as the b-value, the number of measurements, the gradient strength, the echo time, etc., most likely have a direct influence on the uncertainty. It is generally assumed that diffusion data are primarily affected by normally distributed thermal noise, which leads to a Rician distributed signal magnitude. However, physiological noise and artifacts may also affect diffusion data and may result in more complicated and spatially variant noise characteristics.

Several studies report that for the diffusion tensor model, the uncertainty in the tensor trace, diffusion anisotropy, and the tensor major eigenvector are related to the spatial orientation of the tensor (Batchelor et al., 2003; Jones and Pierpaoli, 2004). The orientational dependence of the tensor variance decreases for both more uniformly distributed encoding schemes and increased number of encoding directions. In the experiment using SPARC data, we have demonstrated that the uncertainty of MAP-MRI derived metrics decrease for both increased number of shells and increased number of gradient directions in each shell. The variation in single-fiber area is less sensitive to the number of gradient directions in each shell.

Constraints were not present in original applications of the wild bootstrap (Wu, 1986), thus one concern arises because of the inequality and equality constraints present in Equation (10). A natural question is how the bootstrap approach performs in situations with a constrained parameter space. The bootstrap distribution may be inconsistent with the sampling distribution of the maximum likelihood estimator if the boundary constraints are met (Andrews, 2000). Investigating this issue is beyond the scope of this paper, but further validation studies can be carried out in the future.

In Avram et al. (2016), the computation time for the reconstruction of MAP-MRI parameters from whole-brain diffusion data sets (70 × 70 × 42 × 698) using *N*_max_ = 6 was less than 3 h on a single workstation with 32GB RAM and 8 cores (Intel i7-4770 K at 3.5G Hz). In Fick et al. (2016b), it is reported that it takes around 60 s to do the MAP-MRI fitting (*N*_max_ of 6) for all voxels of SPARC-30, using an Intel(R) Core(TM) i7-3840QM CPU with 32 GB RAM. In this paper, we use two Intel(R) Xeon(R) E5-2697 CPUs and OpenMP to support multi-thread processing, which makes it possible to do the MAP-MRI fitting (*N*_max_ of 6) for all voxels of SPARC-30 within 2 s. To run 500 bootstrap replicates takes about 40 min and 20 h, respectively for the SPARC data and a slice of the HCP-MGH data. In theory, graphics processing units (GPUs) can be used for further speedup (Eklund et al., 2013, 2014) as they can process some 30,000 voxels in parallel.

In conclusion, bootstrap metrics, such as the standard deviation, provide additional valuable information next to the common MAP-MRI parameters, and should be incorporated in ongoing and future MAP-MRI studies to provide more extensive insight.

## Author Contributions

XG and AE contributed conception and design of the study. XG performed the diffusion data analysis, statistical analysis, and wrote the draft of the manuscript. AE, EÖ, and HK provided comments to the manuscript. All authors contributed to manuscript revision, read and approved the submitted version.

### Conflict of Interest Statement

The authors declare that the research was conducted in the absence of any commercial or financial relationships that could be construed as a potential conflict of interest.

## Footnotes

^1^https://github.com/xuagu37/dtb
